# Supplementary figures and images for: FGF and TGFβ signaling link form and function during jaw development and evolution
Source: Dev Biol. Author manuscript; Available in PMC 2019 Dec 1. (PMC6239991; doi:10.1016/j.ydbio.2018.05.002)

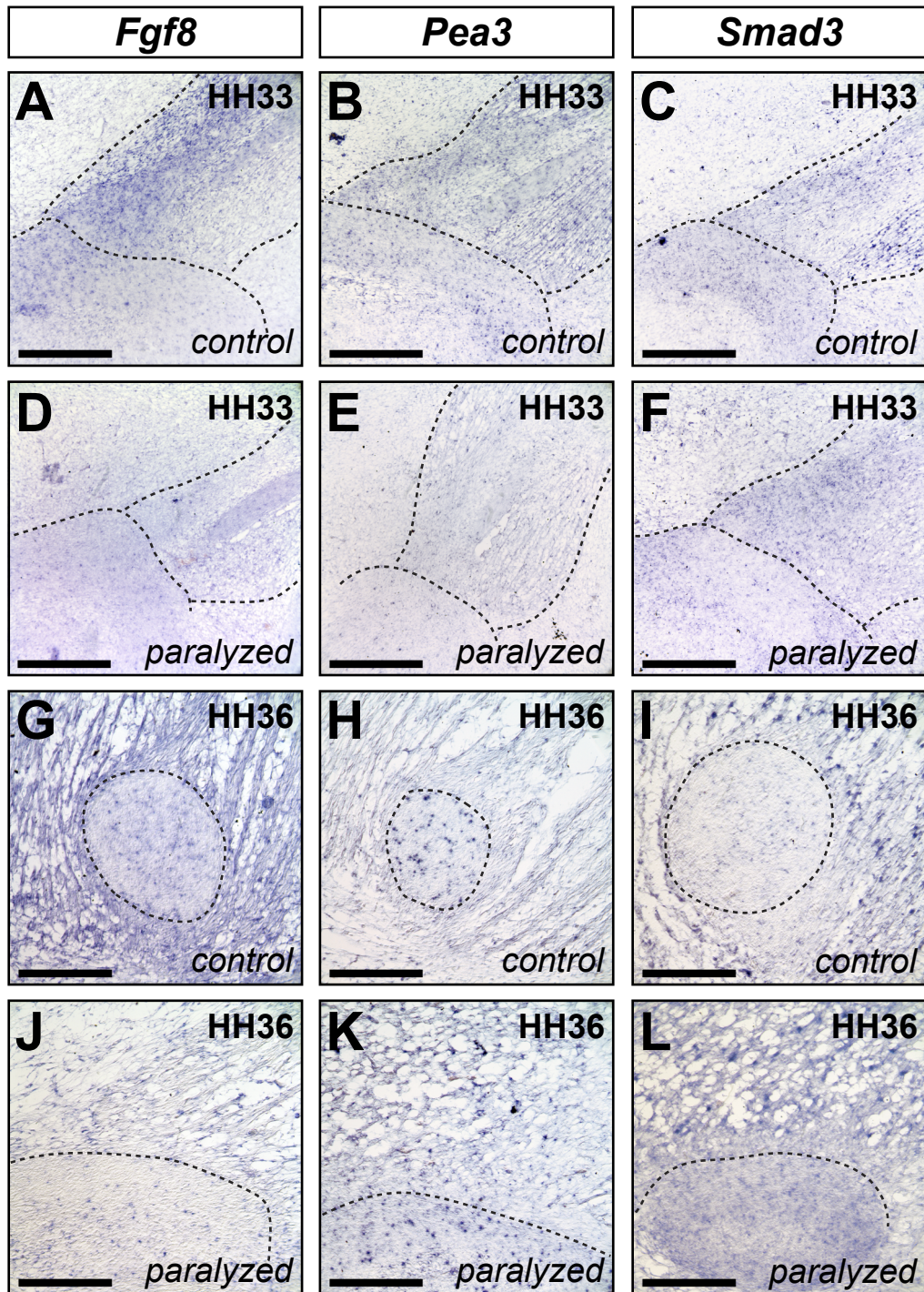

Supplement: 3 [file NIHMS971036-supplement-3.pdf]
