# Supplementary material for: FGF and TGFβ signaling link form and function during jaw development and evolution
Source: Dev Biol. Author manuscript; Available in PMC 2019 Dec 1. (PMC6239991; doi:10.1016/j.ydbio.2018.05.002)
Supplement: TableS1 [file NIHMS971036-supplement-TableS1.pdf]

Supplemental Table 1

Catalog of primers utilized to clone in situ probes and to quantify gene expression

Duck In Situ Primer Pairs

Forward Primer	Reverse Primer
Erm F: *5'*-CTACTGCATCGACTCAGAAG-*3'*	*Erm* R: *5'*-GCCACCTTCTGCATGATG-*3'*
Fgf4 F: *5'*-GCAAACTCTATGGATCTACCCA-*3'*	*Fgf4* R: *5'*-GTGGGAGATACTTTATTGCC-*3'*
Fgf8 F: *5'*-GTGCACGCCAAGCTCAT-*3'*	*Fgf8* R: *5'*-GGTTGAAGGGGTAGTTGAG-*3'*
Fgfr2 F: *5'*-GCTGAAAGATGATGCTACAG-*3'*	*Fgfr2* R: *5'*-CTGAGGTCCAGATACTCCTCGTT-*3'*
Fgfr3 F: *5'*-AAGATGATGCCACAGACAAG-*3'*	*Fgfr3* R: *5'*-ACCCTCCCAAAGTGAAGATC-*3'*
Pea3 F: *5'*-ACATCAAGCAGGAGGTCG-*3'*	*Pea3* R: *5'*-GCCACCTTCTGCATGATGCC-*3'*
Smad3 F: *5'*-CCAGAGAACACTAACTTCC-*3'*	*Smad3* R: *5'*-GGTTCACAGACTGAGCCA-*3'*
Tgfβ2 F: *5'*-AATGCACTGCTATCTCCTG-*3'*	*Tgfβ2* R: *5'*-CAAATCTTGCTTCAGGCTCC-*3'*
Tgfβ3 F: *5'*-CACCGAGTCCGAGTACTATG-*3'*	*Tgfβ3* R: *5'*-CCATAGTCATCCTCACTGTC-*3'*
Tgfβr2 F: *5'*-CTCACAAGAAGAGGAAGCTC-*3'*	*Tgfβr2* R: *5'*-AGCCATGGAGTACACATCTG-*3'*

Duck RT-qPCR Primer Pairs

Forward Primer	Reverse Primer
Erm F: *5'*-GAGACTGGAGGGTAAGGTGAAGC-*3'*	*Erm* R: *5'*-GTCCAGGCGATGAAGTGAGC-*3'*
Fgf2 F: *5'*-GACGGCGTCCGCGAGAAG-*3'*	*Fgf2* R: *5'*-ATTTCAGTGCCAGCAATCTGCC-*3'*
Fgf4 F: *5'*-GCAAACTCTATGGATCTACCCA-*3'*	*Fgf4* R: *5'*-GCATTGTAGTTGTTTGGCAGG-*3'*
Fgf8 F: *5'*-GTGCACGCCAAGCTCAT-*3'*	*Fgf8* R: *5'*-CCTTCTTGTTCATGCAGATGTAGAA-*3'*
Fgfr1 F: *5'*-CTGAAGGAAGGCCACAGGATG-*3'*	*Fgfr1* R: *5'*-TCATGTACAGCTCGTTGGTGCA-*3'*
Fgfr2 F: *5'*-ACCTGCCAACTGCACCAATG-*3'*	*Fgfr2* R: *5'*-CTGAGGTCCAGATACTCCTCGTT-*3'*
Fgfr3 F: *5'*-TGGCCTTGCTAGAGACGTTCAC-*3'*	*Fgfr3* R: *5'*-CACAGGCAGCCGACCATTG-*3'*
MYOD1 F: *5'*-CAACGCCATCCGCTACATCG-*3'*	*MYOD1* R: *5'*-CTGTACTCCATCATGCCGTCG-*3'*
Pai1 F: 5'-AAGAGCGTGGACTTTGAGGA-*3'*	*Pai1 R: 5'*-GATTTCCACAAGCCCTTGAA-*3'*
Pea3 F: *5'*-CTGGACTGGAAGAGGGATGGAG-*3'*	*Pea3* R: *5'*-GCCACCTTCTGCATGATGCC-*3'*
Smad3 F: 5'-CATCCCAGAGACACCTCCTC-*3'*	*Smad3 R: 5'*-GTGTGCCGGAGACATAGGAT-*3'*
Smad7b F: 5'-CCCCCTCCGCCCTACTCCAG-*3'*	*Smad7b R: 5'*-GCCACCACGCACCAGTGTGA-*3'*
SOX9 F: *5'*-AGGGCTCCGAGCAGACCCAC-*3'*	*SOX9* R: *5'*-GCGACTGCCCTGAGTGCTCC-*3'*
Tgfβ2 F: *5'*-TGGCTCCATCACAGAGACAG-*3'*	*Tgfβ2* R: *5'*-CAAATCTTGCTTCAGGCTCC-*3'*
Tgfβ3 F: *5'*-CATCGAGCTCTTCCAGATCC-*3'*	*Tgfβ3* R: *5'*-AAAGTATGGCAAGGGCAGTG-*3'*
Tgfβr1 F: 5'-TGTAGCCACACAAGGCAAAC-*3'*	*Tgfβr1 R: 5'*-TTCCTACTCTGTGGTTGGGG-*3'*
Tgfβr2 F: 5'-GCGAGAGCATCCCTGCGTGG-*3'*	*Tgfβr2 R: 5'*-GCACACCATCTGGATGCCCTGA-*3'*
Tgfβr3 F: 5'-CCGTACAGTGCTTTCCAGGT-*3'*	*Tgfβr3 R: 5'*-TCATGCGACTTGATAACCCA-*3'*
TN-C F: *5'*-CACAGCAGGTGACTCCATGAC-*3'*	*TN-C* R: *5'*-AACACCCTGACTGTGGTTGTTG-*3'*
UCHL-1 F: *5'*-ATTGGTCTGATACACGCAGTTGC-*3'*	*UCHL-1* R: *5'*-TCAACCCGACACTGTCCTTCC-*3'*
β-Actin F: *5'*-ACAGCTTCACCACCACAGCCG-*3'*	*β-Actin* R: *5'*-GCCTCGGGGCACCTGAACCT-*3'*
